# Cloning and Analysis of Expression of Genes Related to Carotenoid Metabolism in Different Fruit Color Mutants of Pepper (*Capsicum annuum* L.)

**DOI:** 10.3390/genes15030315

**Published:** 2024-02-28

**Authors:** Penglong Feng, Yayi Wang, Junqin Wen, Yanjing Ren, Qiwen Zhong, Quanhui Li

**Affiliations:** Academy of Agricultural and Forestry Sciences, Qinghai University/Laboratory for Research and Utilization of Qinghai Tibet Plateau Germplasm Resources, Xining 810016, China; fengpenglong@126.com (P.F.); 2010990020@qhu.edu.cn (Y.W.); 2021990055@qhu.edu.cn (J.W.); 2018990082@qhu.edu.cn (Y.R.); liquanhui_2008@163.com (Q.L.)

**Keywords:** pepper, ripening, carotenoids, gene cloning, expression analyses

## Abstract

The formation of fruit color in pepper is closely related to the processes of carotenoid metabolism. In this study, red wild-type pepper XHB, SP01, PC01 and their corresponding mutants H0809 (orange), SP02 (yellow), and PC02 (orange) were used as research materials. The *Ggps*, *Psy*, *Lcyb*, *Crtz*, *Zep*, and *Ccs* genes involved in carotenoid biosynthesis were cloned, and bioinformatics and expression analyses were carried out. The results showed that the full lengths of the six genes were 1110 bp, 2844 bp, 1497 bp, 2025 bp, 510 bp, and 1497 bp, and they encoded 369, 419, 498, 315, 169, and 498 amino acids, respectively. Except for the full-length *Ccs* gene, which could not be amplified in the yellow mutant SP02 and the orange mutant PC02, the complete full-length sequences of the other genes could be amplified in different materials, indicating that the formation of fruit color in the SP02 and PC02 mutants could be closely related to the deletion or mutation of the *Ccs* gene. The analytical results of real-time quantitative reverse transcription PCR (qRT-PCR) showed that the *Ggps*, *Psy*, *Lcyb*, *Crtz*, and *Zep* genes were expressed at different developmental stages of three pairs of mature-fruit-colored materials, but their patterns of expression were not consistent. The orange mutant H0809 could be amplified to the full *Ccs* gene sequence, but its expression was maintained at a lower level. It showed a significant difference in expression compared with the wild-type XHB, indicating that the formation of orange mutant H0809 fruit color could be closely related to the different regulatory pattern of *Ccs* expression. The results provide a theoretical basis for in-depth understanding of the molecular regulatory mechanism of the formation of color in pepper fruit.

## 1. Introduction

Pepper (*Capsicum annuum* L.) is one of the most widely cultivated vegetable crops worldwide. It is popular among many consumers because its fruit is colorful and has many nutrients, as well as special flavor substances. The pepper fruit ranges in color from immature to mature, including a series of rich fruit colors, such as green, milky white, purple, yellow, and red, among others, which is one of the important commodity traits of pepper. The color of pepper fruit is determined by the types and levels of chlorophyll, carotenoids, and anthocyanins in the pulp cells [1,2]. Anthocyanins and chlorophyll are the primary pigments in immature pepper fruits [3], while carotenoids are the primary pigments in mature pepper fruits [4]. With the development of pepper fruit to physiological maturity, the orange and red carotenoids continue to accumulate, and the chlorophyll and anthocyanins gradually degrade [5]. Red pepper fruit typically contains capsanthin, β-cryptoflavin, β-carotene, capsorubin, zeaxanthin, antheraxanthin, and α-carotene [4,6]. Yellow pepper fruit contains violaxanthin, lutein, α-carotene, β-carotene, zeaxanthin, antheraxanthin, and β-cryptoxanthin, while orange pepper fruit contains capsorubin, β-cryptoflavin, zeaxanthin, violaxanthin, α-carotene, β-carotene, and zeaxanthin [5,7]. The total carotenoid content of red pepper is higher than that of yellow and orange peppers [8,9].

The formation of pepper fruit color is regulated by a series of loci. In immature fruit, purple pepper is controlled by a single dominant gene *A* (Anthocyanin) [10]. The *A* gene is homologous with the Petunia *An2* (Anthocyann2) gene, which is the R2R3 MYB transcription factor gene involved in regulating the biosynthesis of anthocyanin [11]. Milk white and green pepper fruits are controlled by three loci, including *sw1*, *sw2*, and *sw3* [12]. Currently, *APRR2-Like* [13], *CaGLK2* [14], and *CcLOL1* [15] have been identified as genes, which control the color changes in green pepper fruit. In mature pepper fruit, three independent genetic loci (*y*, *c1*, and *c2*) determine the red, yellow, orange, and white color of pepper fruit, respectively [16]. Brown pepper fruit is determined by the *cl* gene [3]. Popovsky [17] confirmed that the color of red fruit is related to the *Ccs* gene, and the *y* locus and *Ccs* gene co-segregate, indicating the unity of the two genes. However, owing to the fact that the red fruit contains capsanthin and capsorubin, the *y* locus is considered to be the gene locus, which controls its synthesis [18,19]. The gene of phytoene synthetase (*Psy*) was found to be co-segregated from the F_2_ population phenotype. It was hypothesized that the *c2* locus controlling the formation of red pepper fruit could be the *Psy* gene, and the gene was located on chromosome 4 [19,20]. The *cl* locus has been determined to be related to the *CaSGR* gene. Typically, the chlorophyll content decreases sharply during the ripening process of pepper fruit with the increases in carotenoid synthesis owing to the transformation of chloroplasts into chromatosomes. However, it has been observed that mutations in the *CaSGR* gene can inhibit the degradation of chlorophyll during the fruit ripening process. In summary, the pepper fruit will be brown (or green) owing to the levels of chlorophyll and carotenoids in the various components [3,21].

Previously, the regulation of biosynthesis of pepper carotenoids has been examined primarily from the viewpoints of structures, the level of expression, and protein products of crucial genes in the carotenoid metabolism pathway. The goal has been to accurately reveal the genetic regulatory mechanism of color changes in mature pepper fruit. Many previous studies have shown that genes such as *Psy* [22], *Crtz* (β-carotene hydroxylase) [23], and *Ccs* (capsaicin synthase) [18] have richly varied structures [24,25]. These variations include gene deletions and mutations in pepper materials with different fruit colors, which lead to the formation of differently colored fruits. Tian [26] used a virus-induced gene signaling (VIGS) method to silence the *Ccs*, *Psy*, *Lcyb* (Lycopene β cyclase), and *Crtz* genes in pepper. The results revealed that the fruit changed from red to yellow or orange. In addition, fruit coloring was observed to be abnormal when multiple genes were silenced simultaneously. After single gene or multiple gene silencing, the levels of capsanthin decreased significantly. In addition, its synthetic substrates, such as β-carotene, β-cryptoxanthin, and zeaxanthin, were also reduced to varying degrees. Kilcrease et al. [25] reported that the increases in total carotenoid content correlated with increases in β-carotene and vioxanthin. The levels of expression of *Psy* and *Crtz-2* were determined to be associated with specific carotenoid contents.

In summary, it has been found that the formation of pepper fruit color has a complex regulatory process. In this study, to further supplement and improve the role of critical structural genes in pepper fruit type carotenoid metabolism pathways during the formation of color, three pairs of red peppers and their mutants were selected as the experimental material. This study cloned, sequenced, and analyzed the expression of critical genes *Ggps* (Geranylgeranyl Pyrophosphate Synthase), *Psy*, *Lcyb*, *Crtz*, *Zep* (Zeaxanthin epoxidase), and *Ccs* in the carotenoid metabolism pathways of samples to provide a theoretical basis for the formation and regulation of different colors in pepper fruit.

## 2. Materials and Methods

### 2.1. Materials

**Figure 1:**
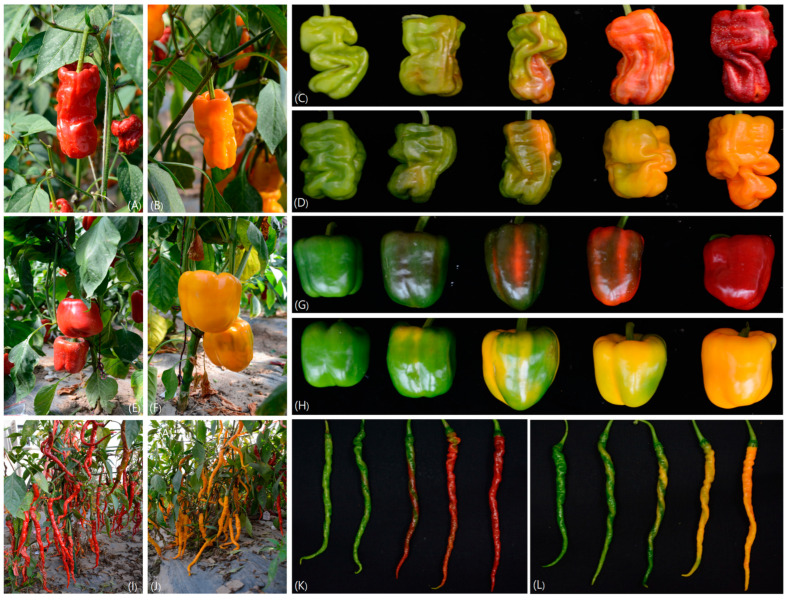
**Experimental material:** (**A**,**B**,**E**,**F**,**I**,**J**) are the field photos of PC01, PC02, SP01, SP02, XHB, and H0809 at the mature stage, respectively. PC01, SP01, and XHB are the wild-type pepper fruits, while PC02, SP02, and H0809 are the corresponding mutants, respectively. (**C**,**D**,**G**,**H**,**K**,**L**) are the images of fruit color at different developmental stages of PC01, PC02, SP01, SP02, XHB, and H0809, respectively.

The test materials were red advanced inbred lines XHB (red), SP01, and PC01 and their mutants H0809 (orange), SP02 (yellow), and PC02 (orange) (Figure 1), which were provided by the Institute of Horticulture, Qinghai Academy of Agriculture and Forestry Sciences (Xining, China). The test materials were sown in 72-hole trays for trial planting in early December 2019 and replanted in a solar greenhouse in March 2020. Based on the different time after anthesis, fruit color changes were divided into five different fruit ripening periods for sampling, including Stage I—green fruit stage, fruit color is green (20 days after anthesis); Stage II—early stage of color change (30 days after anthesis), area of color change accounts for approximately 25%; Stage III—middle stage of color change (40 days after anthesis), area of color change accounts for approximately 50%; Stage IV—late stage of color conversion (50 days after anthesis), area of color conversion accounts for approximately 75%; Stage V—mature stage (60 days after anthesis), area of color conversion is 100%. Uninjured and disease-free fruits were selected when fruit samples were collected. Seeds and placentas were removed, and the pulp was stored at −80 °C after quick freezing with liquid nitrogen. There were three biological repeats for each sample. Healthy leaves were selected to extract the genomic DNA for cloning and an analysis of expression of genes related to carotenoid metabolism.

### 2.2. Extraction and Reverse Transcription of DNA and RNA

The genomic DNA of leaves was extracted using a modified CTAB method [27]. The DNA absorbance values of A260/A280 and A260/A230 were 1.8–2.0 and 2.1–2.3 according to Nanodrop. The RNA of fruits at different periods was extracted using the TaKaRa MiniBEST Plant RNA Extraction Kit (TaKaRa, Dalian, China), and reverse transcription was performed using the PrimeScript^TM^ RT reagent Kit (Perfect Real Time, TaKaRa) according to the manufacturer’s instructions. RNA concentration and quality were determined, and the RNA values of A260/A280 and A260/A230 were between 1.9~2.1 and 2.1~2.3, respectively, which could be used for cDNA synthesis.

### 2.3. Cloning of Carotenoid-Related Genes

Pepper CM334 (http://peppergenome.snu.ac.kr/; accessed on 20 April 2023) was used as the reference genome sequence, and the carotenoid-related genes were cloned via homologous cloning. Primer Premier 5.0 was utilized to design the gene cloning primers (Table 1). With the use of gDNA as a template, the total volume of the PCR reaction system was 50 μL, which included 25 μL of 2 × Prime STAR Max DNA polymerase (TaKaRa), and 0.5 μL of 10 μmol·L^−1^ upstream and downstream primers, respectively. A total of 100 ng of the DNA template and ddH_2_O was added to 50 μL of total volume. The PCR reaction procedure was as follows: 98 °C denaturation for 10 s; 55 °C annealing for 15 s; 72 °C extension for 10 s; and 35 cycles. The PCR product was detected via 1% agarose gel electrophoresis, and the target fragment was gelled and recovered using a centrifuge column agarose gel DNA recovery kit (TIANGEN Biotech Co. Ltd., Beijing, China). Poly A was added to the recovered PCR products using a DNA A-tail Kit (TaKaRa). The total reaction system was 10 μL, which included 10 × A-tail buffer 1 μL; dNTP mixture 0.8 μL; A-tailase 0.1 μL; DNA fragment 100–500 ng; and ddH_2_O added to 10 μL. The reaction procedure was as follows: 72 °C for 15 min and 4 °C heat preservation. The target fragment added with poly A was linked with the sequencing vector pMD18-T (TaKaRa). *Escherichia coli* DH5α competent cells were transformed, and positive mono-clones were selected. The sequencing was performed by Sangon Biotech (Shanghai) Co., Ltd. (Shanghai, China).

### 2.4. Bioinformatics Analysis Process

The BLAST tool in NCBI was used to conduct homologous comparisons and conservative domain analyses of the obtained gene and its encoding protein. The ProtParam tool was used to predict the protein’s molecular weight and theoretical isoelectric point, and DNAMAN 6.0 was used to compare the multiple sequences of amino acids. In addition, MEGA7.0 was adopted to construct a phylogenetic tree, and ProtScale (http://web.expasy.org/cgi-bin/protscale/protscale.pl; accessed on 15 July 2023) was used to analyze the hydrophilicity and hydrophobicity of amino acids. This study also utilized the NetPhos 3.0 server (http://www.cbs.dtu.dk/services/NetPhos/; accessed on 15 July 2023) to predict the protein phosphorylation loci. TMPRED (http://www.ch.embnet.org/software/TMPRED form.html; accessed on 15 July 2023) was used to predict the transmembrane structure, and the SignalP (http://www.cbs.dtu.dk/services/SignalP/;accessed on 15 July 2023) online site was utilized to predict the signal peptide. In addition, WoLF PSORT (https://wolfpsort.org/; accessed on 15 July 2023) was used to predict subcellular localization, and SWISS-MODEL (https://www.swissmodel.expasy.org/; accessed on 15 July 2023) was used to predict the three-dimensional structures of the encoding protein.

### 2.5. qRT-PCR Analysis Process

Quantitative primers were designed according to the cloned gene sequences (Table 1), and the *Ubi3* gene of pepper was used as an internal reference gene [28]. qRT-PCR analysis was performed using the SYBR^®^ Premix Ex Taq II (TaKaRa). A fluorescence quantitative PCR reaction process was performed using an IQ5 Multi-Color Real Time PCR Detection System (Bio-Rad, Hercules, CA, USA) as follows: 95 °C for 3 min; 95 °C for 30 s; 60 °C for 90 s for 40 cycles; and 60 °C for 30 s for 71 cycles, with increases of 0.5 °C for each cycle. Three technical replicates were established for each sample, and the results were analyzed using the 2^−ΔΔCT^ method [29]. SPSS 22.0 (IBM, Inc., Armonk, NY, USA) was adopted for statistical data analysis, and Duncan’s new complex range method was used to complete multiple comparisons.

## 3. Results and Analyses

### 3.1. Cloning and Sequence Analysis of Genes Related to Carotenoid Metabolism

**Figure 2:**
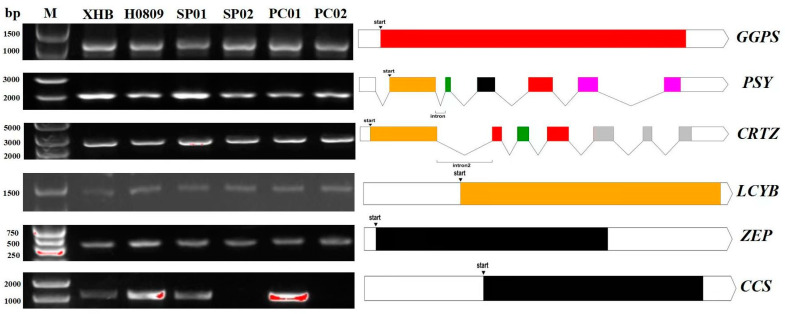
Structures of the key genes involved in the synthesis of carotenoids in pepper.

A homologous cloning strategy was used, which relied on the genome sequence of pepper CM334 and genomic DNA as a template to clone the full-length genes of *Ggps*, *Psy*, *Lcyb*, *Crtz*, *Zep*, and *Ccs* in different mature fruit color materials (Figure 2). The results showed that the full lengths of *Ggps*, *Psy*, *Lcyb*, *Crtz*, *Zep*, and *Ccs* genes were 1110 bp, 2844 bp, 1497 bp, 2025 bp, 510 bp, and 1497 bp, and they encoded 369, 419, 498, 315, 169, and 498 amino acids, respectively. An analysis of the gene structure showed that *Ggps*, *Lcyb*, *Zep*, and *Ccs* had no intron sequence; *Psy* contained six exons and five introns; and *Crtz* contained seven exons and six introns. A sequence comparison with related genes in the reference genome CM334 showed that there was one base difference (G/A) at 1437 bp of the *Crtz* gene, but in our selected experimental materials, the sequence of the *Crtz* gene was completely consistent.

The results of sequence alignment among different materials showed that the sequences of other genes were completely consistent among the different cultivars, except that *Ccs* exhibited sequence differences among the different mutant materials. *Ccs* could be amplified in the three red wild-type pepper materials. Among the three mutants, only the complete *Ccs* full-length sequence could be amplified in H0809 but not in the other two mutants, indicating that the formation of fruit color in SP01 (yellow) and PC02 (orange) could be closely related to the deletion or mutation of *Ccs*.

### 3.2. Bioinformatics Analysis of Genes Related to Carotenoid Biosynthesis

The physical and chemical properties of Ggps, Psy, Lcyb, Crtz, Zep, and Ccs proteins were analyzed using ExPASy ProtParam (Table 2). The pIs of most proteins exceeded 7.0, with the exception of Ggps and Lcyb. All proteins but Zep had at least 315 total amino acids, and Crtz was the only protein, which had a positive hydrophilicity value.

**Figure 3:**
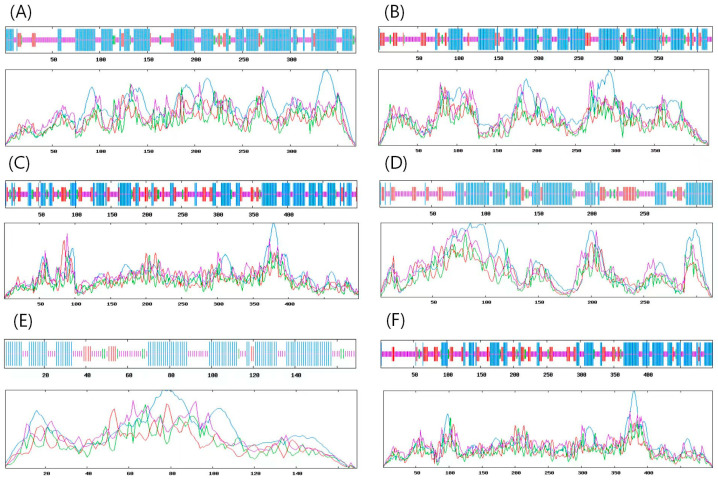
Prediction of proteins’ secondary structures: (**A**–**F**) represent the secondary structures of Ggps, Psy, Crtz, Lcyb, Zep, and Ccs proteins, respectively. Blue indicates an α-helix; red denotes the extended strand; green is a β-fold; and purple indicates a random coil.

The PSIPRED online software was used to predict the secondary structures of proteins in this study (Table 3, Figure 3). α-Helix and random coils were the primary structural elements constituting the secondary structures of proteins in the Ggps, Psy, Lcyb, Crtz, Zep, and Ccs proteins, and they accounted for more than 70%, as shown in Table 3. The signal peptide encoded by the SignalP3.0 online software predicted that Ggps, Lcyb, Crtz, Zep, and Ccs had no signal peptides. However, Psy contained a signal peptide with 20 amino acid residues, and its score was 0.342. Therefore, based on the results of an analysis using Plant-mPLoc software, the Zep, Lcyb, Ccs, and Psy proteins were localized in the chloroplasts. Ggps was localized in the chloroplasts or plastids, and Crtz proteins were localized in the vacuoles.

**Figure 4:**
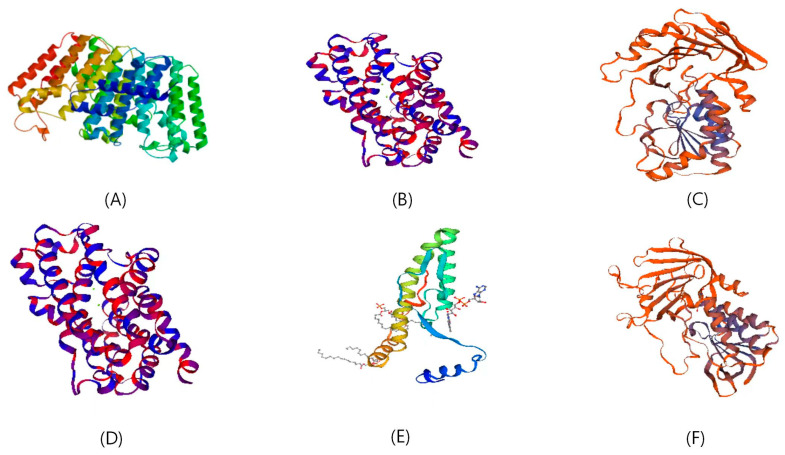
Tertiary structures of protein products encoded by genes related to carotenoid synthesis in pepper. (**A**–**F**) represent the tertiary structures of Ggps, Psy, Crtz, Lcyb, Zep, and Ccs proteins, respectively.

The possible configurations of protein tertiary structures were predicted using the SWISS-MODEL online software and were found to be consistent with the predicted protein secondary structures (Figure 4).

### 3.3. Evolutionary Analysis

**Figure 5:**
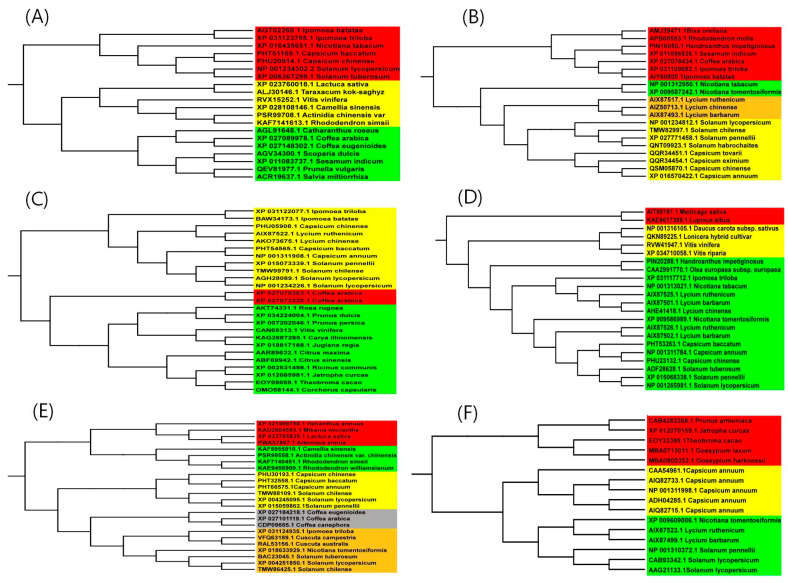
Evolutionary tree analysis of the amino acid sequences of genes related to carotenoid synthesis in pepper. (**A**–**F**) represent the evolutionary trees of *Ggps*, *Psy*, *Crtz*, *Lcyb*, *Zep*, and *Ccs* genes, respectively.

In order to study the evolutionary relationship of genes related to carotenoid synthesis in pepper, a phylogenetic tree was constructed using the neighbor-joining method in MEGA7.0 (Figure 5). The results showed that *Ggps* of the different pepper varieties belonged to the same branch as that of tomato (*Solanum lycopersicon*), which was distantly related to the evolution of periwinkle (*Vinca minor*) and coffee (*Coffea* spp.), among others. *Psy* was found to have a close evolutionary relationship with pepper, tomato, and wolfberry (*Lycium barbarum*). *Lcyb*, *Crtz*, and *Ccs* of pepper were observed to have a close evolutionary relationship with tomato but a distant evolutionary relationship with coffee and rose (*Rosa* spp.). The *Zep* gene of pepper was not only closely related to the evolution of different varieties of pepper and tomato but also closely related to the evolution of different varieties of coffee. In general, the evolution of genes related to carotenoid synthesis in pepper is in the same branch as that of other solanaceous crops, such as tomato and potato (*Solanum* *tuberosum*).

### 3.4. Homology Analysis

**Figure 6:**
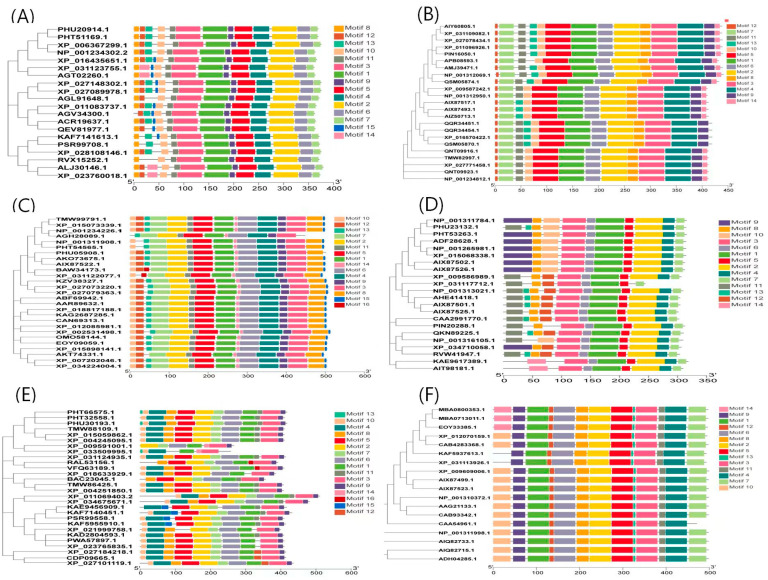
Conservative motifs: (**A**–**F**) represent the conserved motifs of Ggps, Psy, Crtz, Lcyb, Zep, and Ccs proteins, respectively.

The MEME online software and TBtools software were used to visualize conservative motifs. The results showed that the closer the relatives are in the phylogenetic tree, the more similar or identical the conserved motifs contained in the Ggps, Psy, Lcyb, Crtz, Zep, and Ccs proteins are (Figure 6). There were 13 identical conserved motifs (Motifs 1–13) in the Ggps proteins in Solanaceae crops with close relatives (*Capsicum chinense* Jacq: PHU20914.1, *Capsicum annuum*: PHT51169.1, potato: XP_0063672991.1, and tomato: NP_001234302.2). The Psy protein also had 13 identical conserved motifs (Motifs 1–13) in different varieties of pepper (*Capsicum annuum*: QQR34451.1, *Capsicum eximium* Hunz.: QQR34454.1, *Capsicum annuum*: XP_016570422.1, and *Capsicum chinense* Jacq: QSM05870.1). The Zep, Ccs, Lcyb, and Crtz proteins also only had 1–2 differences in the conserved motifs among different species, which indicated that the sequences of these genes were relatively conserved in both pepper and other species.

### 3.5. qRT-PCR Analysis

**Figure 7:**
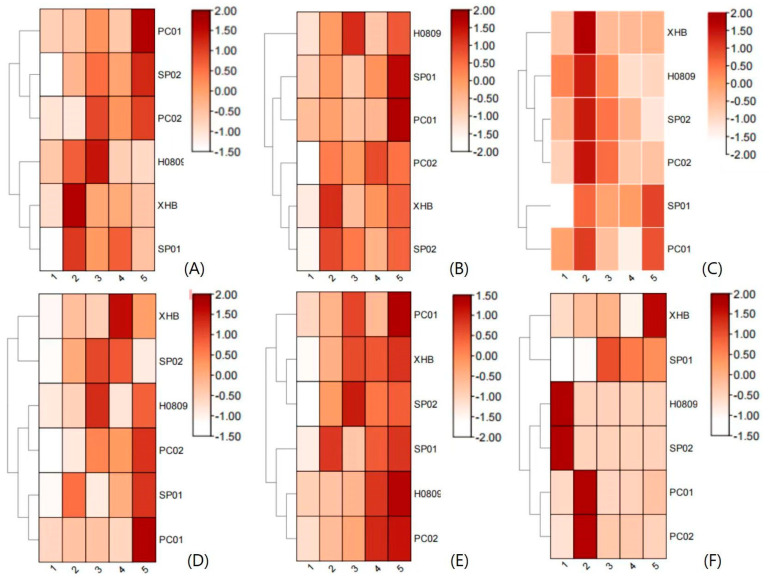
Analysis of the expression of genes related to carotenoid synthesis in pepper. (**A**–**F**) exhibit the expression heatmaps of genes representing *Ggps*, *Psy*, *Crtz*, *Lcyb*, *Zep*, and *Ccs*, respectively.

The expression patterns of *Ggps*, *Psy*, *Lcyb*, *Crtz*, *Zep*, and *Ccs* genes in different fruit color materials and different fruit ripening stages were analyzed using qRT-PCR (Figure 7). The results showed that with the increase in fruit maturity, the expression of *Ggps* in XHB decreased gradually, and the expression was highest at Stage II. In the yellow wild-type H0809, the amount of expression first increased and then decreased, and the amount of expression was highest at Stage III. In SP02 and PC02, *Ggps* was more highly expressed than in its red wild type.

In XHB, the level of expression of *Psy* was highest at Stage II and lowest at Stage III. In H0809, the expression of *Psy* was higher than that of the wild-type XHB and reached its highest level at Stage III. The level of expression of *Psy* in SP01 was higher than that in its yellow mutant SP02. In SP01, the amount of expression was highest in Stage V. In SP02, the level of expression of *Psy* gradually decreased, while its level of expression increased slightly at Stage V. In PC01, *Psy* was primarily expressed at Stage V, and in PC02, except for Stage V, the level of expression of *Psy* was significantly higher than that of the wild-type PC01.

Except for the higher level of expression of the *Crtz* gene at Stage IV of XHB and Stage V of SP01, the level of expression of the *Crtz* gene in mutants was generally higher than that of the red wild type. The expression of model gene *Lcyb* in all the materials was basically the same. Except for SP02, the amount of expression reached its maximum at Stage II. In addition to the higher level of expression of XHB at Stage II, the level of expression of *Zep* in mutant materials was higher than that in the red wild type. The pattern of expression of *Zep* in all the other materials increased overall and reached its maximum at fruit maturity.

In terms of different materials, the *Ggps*, *Lcyb*, and *Ccs* genes were highly expressed at each stage of red pepper XHB, and the *Psy*, *Crtz*, and *Zep* genes were highly expressed in H0809. In sweet pepper SP01, the *Psy*, *Lcyb*, and *Ccs* had higher levels of expression, while in the yellow mutant SP02, *Ggps*, *Crtz*, and *Zep* had higher levels of expression. In PC01, only the level of expression of the *Ccs* gene was higher, and the overall expression of *Ggps*, *Psy*, *Crtz*, *Lcyb*, and *Zep* genes in PC02 was higher than that in PC01. It can be deduced that the patterns of expression of genes related to carotenoid metabolism are not consistent in pepper materials with different fruit colors.

The expression of the *Ccs* gene was relatively high in the red wild-type materials and reached its maximum at Stages IV and V. The *Ccs* gene was not expressed in SP02. In the orange mutant H0809, the expression of the *Ccs* gene was maintained at a low level at different developmental stages of fruit color, which was significantly lower than that of the wild-type XHB, indicating that the formation of fruit color of orange mutant H0809 and yellow SP02 could be closely related to different levels of expression and regulatory modes of *Ccs*.

## 4. Discussion

Carotenoids are the primary pigments in the flowers and fruits of most plants. During the development of pepper fruits, the types and contents of carotenoids in different types of colored fruits vary. The composition and content of carotenoids affect the color of pepper fruits [30,31,32,33]. Currently, the regulation of carotenoid synthesis in pepper has been primarily focused on the structures, levels of expression, and protein products of *Psy*, *Lcyb*, *Lcye*, *Crtz*, and *Ccs* genes, with the goals of revealing the genetic regulatory mechanisms of changes in the ripening fruit color of pepper [4,34,35]. The results of this study showed that among the three pairs of different mutant materials, only the *Ccs* gene was diverse in different materials, and the sequences of other genes were completely consistent. The results of a qRT-PCR analysis showed that the patterns of expression of the six genes described above in different materials were not consistent, indicating that there could be complex regulatory mechanisms in the process of fruit color formation.

Currently, there are few reports on the role of *Ggps* in the formation of pepper fruit color. Geranylgeranyl pyrophosphate (GGPP) is the most direct precursor of carotenoids [36]. *Ggps* has been found to play an essential role in carotenoid synthesis and accumulation in some plants [37,38]. It has been observed that overexpression of the *Ggps* gene in brewer’s yeast will significantly increase the content of β-carotene [39]. Overexpression of *Ggps* also significantly increased carotenoid production in yeast *Phaffia rhodozyma* [40,41]. Therefore, in plants, *Ggps* is often a polygene family compound, and its proteins are expressed in different subcellular structures [42]. Previous studies on *Ggps* in pepper have localized it in the plastid septum, and induced levels of expression were observed during the fruit ripening stages [43]. This study cloned the full-length *Ggps* gene, which encoded 291 amino acids from three groups of mutant materials with different fruit colors. There was no difference in the sequence of *Ggps* gene between the different materials. Subcellular localization predicted that *Ggps* was located in the chloroplast or plastid, which was consistent with previous research.

*Psy* is the primary regulatory enzyme in the carotenoid synthesis pathway, which also makes the *Psy* gene one of the first-choice genes for improved plant breeding [20]. There are two *Psy* genes in pepper [19,25]. Recent research results have shown that *Psy1* has the highest levels of expression in the fruit pericarp and petals, and the *Psy2* gene was detected in all organs analyzed. Its level of expression was highest in cotyledons and lowest in the pericarp. It was detected not only in the leaves and immature fruit but also in mature fruit [44]. Some studies also found that *Psy2* could compensate for the loss of *Psy1* in pepper fruit [45]. In this study, only the *Psy1* gene was cloned, and the patterns of expression were analyzed at different stages of fruit ripening. The results showed that there was no difference in the sequence of *Psy1* among different pepper materials, but its pattern of expression was very different. Typically, the gene was expressed more highly during the fruit color transformation and ripening stages, which was basically consistent with the conclusions of previous research.

In pepper, mutations at base 709 of the *β-CHY2* (*β-carotene hydroxylase 2*) gene in the ethane methanesulfonate (EMS)-induced orange fruit mutants resulted in the content of β-carotene in orange fruit being 3.2-fold higher than that in red fruit, while the total amount of carotenoids was reduced by 58.3% [23]. Overexpression of the *β-CHY* gene in other crops contributes to the accumulation of zeaxanthin in cells, thereby increasing the content of carotenoids in cells [46]. The *Arabidopsis* mutants, which inhibit the expression of *β-CHY* via antisense suppression and T-DNA mutation, have a weakened ability to synthesize cryptoxanthin and other carotenoids, and their resistance to stress significantly decreases [47,48].

There are two genes encoding the *Lcyb* gene in pepper plants. *Lcyb1* plays a role in the β-ε-carotene branch, while *Lcyb2* plays a role in the β-carotene branch [49,50]. A single-base mutation in the coding region of *Lcyb* in papaya (*Carica papaya*) affects enzyme activity, resulting in red-fleshed papayas, which accumulate lycopene in the fruit [51]. In addition, *Lcyb2* in saffron (*Crocus sativa*) plays a decisive role in its stigma color [52]. In this study, there was no difference in the sequence of *Lcyb* in different materials, but the patterns of expression were inconsistent. *Zep* catalyzes the conversion of zeaxanthin to antherxanthin, which in turn produces vioxanthin [53]. Studies on sweet potato (*Ipomoea batatas*) found that the *ZEP* paralog *g1103.t1* could be involved in the accumulation of carotenoids owing to the epoxidation of β-carotene and β-cryptoxanthin [54]. In this study, there was no difference in the sequence of *Lcyb* and Zep in different materials, but the patterns of expression were inconsistent.

*Ccs* encodes capsanthin/capsanthin synthetase, which has a rich structural diversity. Deletion of *Ccs* or loss of function owing to its sequence variation results in the formation of yellow- or orange-fruited peppers [24,25,55]. Several studies confirmed the deletion of 211 and 220 bp at the 3’ end of the *Ccs* gene in yellow-fruited peppers [17,56]. It was also reported that a frameshift mutation in the *Ccs* coding region of orange-fruited annual pepper ‘Fogo’ caused premature termination of transcription, and its fruit primarily contained β-carotene and zeaxanthin but did not contain capsanthin [5]. Similar reports showed that in the yellow fruit pepper ‘CK7’, a single-base (C/G) mutation occurred in the *Ccs* gene at 1095 bp downstream of the transcription start site, resulting in formation of a premature stop codon [57]. In yellow-fruited pepper ‘Y2,’ the *Ccs* gene was inserted 8 bp at 1431 bp, resulting in a frameshift mutation and premature termination of transcription. ‘Y3’ had a single-base (C/A) mutation at 599 bp. Therefore, it was assumed that variation in the *Ccs* gene was one of the reasons for the formation of yellow and orange mutants.

The results of this study also confirmed that the *Ccs* genes are also diverse in the different fruit-colored pepper materials used in this study. In the two mutant materials SP02 (yellow) and PC02 (orange), we were unable to amplify the wild-type *Ccs*. However, we were able to amplify the red XHB, SP01, and PC01 and orange mutant H0809 to the full-length sequence of the *Ccs* without sequence differences. Expression of the *Ccs* in H0809 only remained at a low level, and the difference was not significant at different developmental stages. Thus, we can hypothesize that the formation of orange pepper H0809 fruit color may not be due to *Ccs*. In addition to the mutation of genes, their differential regulatory patterns could lead to the formation of orange mutant fruit color, and its precise regulatory mechanism merits further study. No transcription of *Ccs* was detected in SP02, but in PC02, it was expressed to varying degrees. It was hypothesized that the formation of fruit color in SP02 could be caused by deletion of the *Ccs* gene, and there could be homologous sequences of the *Ccs* gene in PC02, which merits further study.

## 5. Conclusions

In this study, three pairs of different mature fruit color mutants of peppers were used as research materials to perform cloning, bioinformatics, and expression analyses of the *Ggps*, *Psy*, *Lcyb*, *Crtz*, *Zep*, and *Ccs* genes related to carotenoid biosynthesis. The results of this study showed the following:

(1) In both wild-type XHB and its orange mutant H0809, we were able to amplify the complete *Ccs* gene. However, the level of *Ccs* expression remained at a low level in mutant H0809, and the difference in expression was significant compared with that of the wild-type XHB, indicating that the formation of fruit color of the orange mutant H0809 could be closely related to the different regulatory patterns of expression of *Ccs*.
(2) Compared with H0809, we could not amplify the complete *Ccs* gene in mutants SP02 and PC02, and at the same time, we could not detect the transcript product of the *Ccs* gene in SP02, but the transcript product could be detected in PC02, indicating that the formation of mutant SP02 may be related to deletion of the *Ccs* gene, and there may be a homolog of the *Ccs* gene in PC02.
(3) We were able to amplify the complete *Ggps*, *Psy*, *Lcyb*, *Crtz*, and *Zep* genes in three different pairs of wild-type and mutant materials, but their expression patterns were inconsistent across the different developmental stages of different materials. Overall, the expression levels of *Psy* and *Crtz* genes in different materials were relatively high, and the expression levels of *Ccs* genes in red wild type were generally higher than those in mutant materials.

## Figures and Tables

**Table 1:** Sequence information of the related primers required for the experimental processes.

Primer Name	Primer Sequence (5′-3′)	Purpose	Product Size (bp)	Tm (°C)
Ggps-F/R	GAACCTTGTTGATTTATGGGC CCAACATAAGCACACTGAAAG	PCR amplification	1110	57
Psy-F/R	ATGTCTGTTGCCTTGTTATGG CCTGATTTCATGTTCTTGTAGAAGG	PCR amplification	2844	58
Lcyb-F/R	ATGGATACGCTCTTGAGAACCCCAAAC TCATTCTTTATCCTGTAACAAATTGTT	PCR amplification	1497	58
CRTY-F/R	ATGGCTGCTGAAATTTCAATCTCCG CATAATCTCTTCGAACTTTTAATTC	PCR amplification	2024	58
Zep-F/R	ATGAAGCAATTTGTGCTAAGTTTGG TCACTTTTGACATAATTTTCCGCAA	PCR amplification	510	57.5
Ccs-F/R	TCTCTAATGGAAACCCTTCTAAAGCC TCAAAGGCTCTCTATTGCTAGATTGC	PCR amplification	1497	58
RT-*Ggps*-F/R	CATTGTCAACTCCACGGC CCGTAGATTTTGTGGTTGGT	qRT-PCR	158	60
RT-*Psy*-F/R	ACAGGCAGGTCTATCCGACGAAG ACAACAGCAGAGATGCCAACACAG	qRT-PCR	164	60
RT-*Lcyb*-F/R	GTTGTTGGAATTGGTGGCACAGC ATGGCATTGGCAACGACAGGAG	qRT-PCR	182	60
RT-*CRTY*-F/R	GCACGAGTCACACCATAGACCAAG CGTGAACGAACATGTAGGCCATCC	qRT-PCR	155	60
RT-*Zep*-F/R	TGCACTTCATCCAATGACACCT GCCTCTGAAATGCACCTTGC	qRT-PCR	164	60
RT-*Ccs*-F/R	AGCACCCACATCAAAGCCAG GTGGTGAAGGGTCAACGCAA	qRT-PCR	145	60
Ubi3-F/R	TGTCCATCTGCTCTCTGTTG CACCCCAAGCACAATAAGAC	Internal reference	155	60

Note: qRT-PCR, real-time quantitative reverse transcription PCR; F, forward primer; R, reverse primer.

**Table 2:** Physicochemical properties of proteins related to the synthesis of pepper carotenoids.

Gene Name	Total Amino Acids	Molecular Weight of the Protein	Molecular Formula	Isoelectric Point	Coefficient of Instability	Positively Charged Amino Acids	Negatively Charged Amino Acids	Total Hydrophilicity Value
Ggps	369	40,173.41	C_1780_H_2889_N_485_O_536_S_16_	6.12	37.01	43	47	−0.012
Psy	419	47,066.05	C_2083_H_3320_N_588_O_614_S_20_	9.01	53.5	60	52	−0.242
Lcyb	498	55,626.23	C_2498_H_3930_N_668_O_719_S_25_	6.34	37.73	54	58	−0.073
Crtz	315	35,317.91	C_1612_H_2475_N_431_O_434_S_15_	8.52	54.43	35	32	0.012
Zep	169	18,995.24	C_842_H_1371_N_227_O_246_S_12_	8.95	38.15	24	18	−0.073
Ccs	498	56,658.66	C_2549_H_4007_N_689_O_718_S_27_	8.77	44.15	66	58	−0.216

**Table 3:** Predictions of the secondary structures of gene proteins related to carotenoid synthesis in pepper.

Protein Name	α-Helix	Extension	Β-Turn	Random Coil
Ggps	54.74%	6.5%	5.15%	33.6%
Psy	51.79%	12.17%	3.82%	32.22%
Lcyb	39.56%	16.87%	6.02%	37.55%
Crtz	45.08%	15.24%	5.4%	34.29%
Zep	56.21%	5.92%	4.73%	33.14%
Ccs	34.54%	17.27%	4.02%	44.18%

## Data Availability

The original contributions presented in the study are included in the article, further inquiries can be directed to the corresponding authors.
